# Estimating vaccine effectiveness against SARS-CoV-2 infection, hospitalization and death from ecologic data in Costa Rica

**DOI:** 10.1186/s12879-022-07740-5

**Published:** 2022-10-02

**Authors:** Romain Fantin, Rolando Herrero, Allan Hildesheim, Cristina Barboza-Solís, Amada Aparicio, D. Rebecca Prevots, Ruth M. Pfeiffer, Mitchell H. Gail, the RESPIRA Study Group

**Affiliations:** 1 Centro Centroamericano de Población, Universidad de Costa Rica, San José, 2060 Costa Rica; 2 Agencia Costarricense de Investigaciones Biomédicas, Fundación INCIENSA, San José, Costa Rica; 3 Facultad de Odontología, Universidad de Costa Rica, San José, Costa Rica; 4 Centro de Desarrollo Estratégico e Información en Salud y Seguridad Social (CENDEISSS) Caja Costarricense de Seguro Social, San José, Costa Rica; 5 Epidemiology and Population Studies Unit, Division of Intramural Research, National Institute of Allergy and Infectious Diseases, Rockville, MD USA; 6 Biostatistics Branch, Division of Epidemiology and Genetics, National Cancer Institute, Bethesda, MD USA; 7 Division of Cancer Epidemiology and Genetics, National Cancer Institute, 9609 Medical Center Drive RM 7-E138, MSC 9780, Bethesda, MD 20892 USA

**Keywords:** Vaccine effectiveness, Ecologic estimate of vaccine effectiveness, SARS-CoV-2, COVID-19

## Abstract

**Background:**

Clinical trials and individual-level observational data in Israel demonstrated approximately 95% effectiveness of mRNA-based vaccines against symptomatic SARS-CoV-2 infection. Individual-level data are not available in many countries, particularly low- and middle- income countries. Using a novel Poisson regression model, we analyzed ecologic data in Costa Rica to estimate vaccine effectiveness and assess the usefulness of this approach.

**Methods:**

We used national data from December 1, 2020 to May 13, 2021 to ascertain incidence, hospitalizations and deaths within ecologic units defined by 14 age groups, gender, 105 geographic areas, and day of the epidemic. Within each unit we used the proportions of the population with one and with two vaccinations, primarily tozinameran. Using a non-standard Poisson regression model that included an ecologic-unit-specific rate factor to describe rates without vaccination and a factor that depended on vaccine effectiveness parameters and proportions vaccinated, we estimated vaccine effectiveness.

**Results:**

In 3.621 million persons aged 20 or older, there were 125,031 incident cases, 7716 hospitalizations, and 1929 deaths following SARS-CoV-2 diagnosis; 73% of those aged ≥ 75 years received two doses. For one dose, estimated effectiveness was 59% (95% confidence interval 53% to 64%) for SARS-CoV-2 incidence, 76% (68% to 85%) for hospitalizations, and 63% (47% to 80%) for deaths. For two doses, the respective estimates of effectiveness were 93% (90% to 96%), 100% (97% to 100%), and 100% (97% to 100%).

**Conclusions:**

These effectiveness estimates agree well with findings from clinical trials and individual-level observational studies and indicate high effectiveness in the general population of Costa Rica. This novel statistical approach is promising for countries where ecologic, but not individual-level, data are available. The method could also be adapted to monitor vaccine effectiveness over calendar time.

**Supplementary Information:**

The online version contains supplementary material available at 10.1186/s12879-022-07740-5.

## Background

Vaccines against SARS-CoV-2 (or COVID-19) were effective in preventing infections, hospitalizations and deaths early in the pandemic. Clinical trials of the mRNA-based vaccines BNT162b2 (Pfizer-BioNTech or tozinameran) and mRNA-1273 (Moderna) demonstrated approximately 95% efficacy against symptomatic infection [1, 2]. To assess “real world” effectiveness in the general population, observational studies have been conducted in Israel [3], Qatar [4, 5], Ontario, Canada [6], the US [7], and possibly elsewhere. The study of tozinameran in Israel [3] followed vaccinated and unvaccinated people through record linkage and reported effectiveness estimates of 97.0% against symptomatic COVID-19 infection, 97.2% against COVID-19-related hospitalization, and 96.7% against COVID-19-related death. The other studies used case–control designs and also demonstrated high vaccine effectiveness. All these studies used individual-level data on vaccination and health outcomes. In many countries, such individual-level data would not be available, either because of confidentiality restrictions, lack of resources to conduct large cohort or case–control studies, or absence of needed medical record systems.

Using a novel Poisson regression model, we estimated vaccine effectiveness in the general population of Costa Rica from an ecologic analysis of the association of proportions vaccinated in ecologic units with COVID-19 incidence and with related hospitalizations and deaths in those units. The ecologic units were defined by age group, gender, geographic area, and day of the epidemic from December 1, 2020 to May 13, 2021. During this period, the delta variant was not identified in Costa Rica, 97% of those vaccinated received tozinameran, and 3% received COVISHIELD (Oxford/AstraZeneca formulation). This approach covers the entire population of Costa Rica and may be feasible in other countries where individual-level studies are not.

## Methods

### Study design and population

We analyzed nationwide surveillance data from December 1, 2020 to May 13, 2021 for the 3.62 million members of the Costa Rican population aged 20 years and older. Data on COVID-19 incidence, hospitalizations and death were obtained from public online files from the national Ministry of Health. Data on vaccinations were provided by request from the Costa Rican National Social Security Fund (Caja Costarricense del Seguro Social or CCSS).

Ecologic units were defined jointly by 14 age groups (20–24, 25–29, …, 80–84, 85 +), gender (male, female), 105 health areas (*Áreas de Salud* or *AS*) and day of the epidemic, beginning with December 1, 2020. National data for the number of native-born Costa Ricans in each age-by-gender group were reported for the 488 districts of Costa Rica by the Tribunal Supremo de Elecciones (Electoral Court), based on tracking the unique identification number (cédula) given at birth. These age-, gender- and district-specific counts were increased to allow for persons born outside Costa Rica by multiplying by the age- and gender-specific ratios of native- and non-native-born persons to native-born persons found from the 2011 Census. Twenty-eight percent of the districts fall into more than one *AS*, but, if so, the vast majority of the population is in one *AS* typically. The age- and gender-specific populations of such districts were allocated to the several *AS* using the overall proportions of the population in each *AS* provided by the CCSS. We cross-checked these population estimates against reported COVID-19 vaccination data. If the reported number of vaccinated people in August 2021 exceeded the census estimate of the population in a specific age, gender and *AS* category, the population was set to the number reported vaccinated. This happened in 13% of the age × gender × *AS* categories. To preserve the national age- and gender-specific census estimates, the people added by the vaccination adjustment in a given *AS* were removed proportionally from the other *AS* for that age and gender category.

### Incidence data on cases, hospitalizations and deaths with COVID-19

The Ministry of Health reported the number of incident COVID-19 cases by age group, gender, district and day of diagnosis. Cases in a district that spanned more than one *AS* were allocated as described above for the district population. A case was diagnosed either by a positive PCR test (82% of the diagnoses) or, beginning in August 2020, by reporting symptoms and living with a person who received a positive PCR test [8] (18% of the diagnoses [9]). Hospitalizations and deaths from any cause were assigned the date of the COVID-19 diagnosis and were based on reports of hospitalizations and deaths through July 5, 2021.

### PCR testing for SARS-CoV-2

The CCSS, that provides health services to the vast majority of the population in Costa Rica, in collaboration with the Ministry of Health, was the first source of PCR testing, with later expansion to private laboratories. Testing is provided free of charge. Testing has been used mainly to confirm the diagnosis of symptomatic cases. Travelers and a limited number of workers in some industries underwent screening when asymptomatic, as did patient contacts in the early phase of the pandemic when the number of cases was small. During the period included in this study, testing was available to the entire CCSS population with few exceptions.

### Vaccination data

The CCSS provided vaccination data aggregated into categories defined by age group, gender, *AS* and day of the epidemic. For each day, the number of people who had previously received only a first dose 14 or more days before and the number of people who had received two doses at least 14 days before was reported. In 4.5% of the ecologic units, the number of people reported to have received the second dose was slightly greater than the number of people reported to have received only the first dose, in which case we replaced the number reported as having received only a first dose 14 or more days earlier with the number reported as having received a second dose 14 or more days earlier.

COVID-19 vaccination in Costa Rica started in December 2020, and the distribution of vaccine was strictly controlled by the Ministry of Health and the CCSS. Vaccination is not available for purchase in the private sector and is only offered at CCSS clinics and hospitals. Thus, allocation of vaccine to targeted groups has been strictly enforced. The first targeted group consisted of subjects over 58 years of age with risk factors for severe disease (e.g., hypertension, diabetes, immunosuppression, advanced age). Due to vaccine shortages, vaccination of this first group only accelerated beginning in early March 2021. The second targeted group (beginning end of February 2021) were subjects over 58 years old regardless of risk factors. Younger adults were eligible after April 28, 2021. The addition of a new targeted group was authorized in a specific *AS* when 80% of the previous targeted group was vaccinated.

### Informed consent

We used de-identified grouped data for which informed consent is not required.

### Statistical analysis

We plotted the proportions of the population vaccinated as a function of time for various age groups. We also plotted on a logarithmic scale the seven-day average numbers of incident cases, cases that were hospitalized, and cases that died against the day, *d*, of case diagnosis. The average included counts from days *d*, *d* − 1, …, *d* − 6. Based on the model described below, we plotted the numbers of deaths among COVID-19 cases in the presence and in the hypothetical absence of the vaccination program.

We used a novel Poisson regression model to estimate vaccine effectiveness, based on the observed events in each ecologic unit ${Y}_{a,s,d,AS}$. Here the event is COVID-19 incidence, hospitalization or death. Separate models were developed for each of these outcomes. The expected value of ${Y}_{a,s,d,AS}$ in the regression is

1 $$E\left( {Y_{a,s,d,AS} } \right) = population_{a,s,AS} \times \exp \left( {X\beta } \right) \times \left( {1 - \rho_{1} p_{1,a,s,d,AS} - \rho_{2} p_{2,a,s,d,AS} } \right)$$

where ${population}_{a,s,AS}$ is the population size, which is assumed constant over time, ${p}_{1,a,s,\mathrm{d},AS}$ is the proportion of the population in the ecologic unit that has received only one dose of the vaccine at least 14 days before day *d*, ${p}_{2,a,s,\mathrm{d},AS}$ is the proportion that has received two doses at least 14 days before day *d*, and ${\rho }_{1}$ and ${\rho }_{2}$ are the respective vaccine effectiveness parameters. The effectiveness of a vaccine dose is defined as one minus the corresponding relative risk that compares vaccinated to unvaccinated subjects [10]. The factor $r_{a,s,d,AS} = \exp \left( {X\beta } \right)$ represents the event rate in the absence of vaccination for the ecologic unit defined by $\left( {a,s,d,AS} \right)$. The calendar time effect is modeled as a sixth-degree polynomial for each of the seven health regions of Costa Rica in which the *AS* reside. The model also includes day of the week. Further details on derivation of the expectation equation, on the function $\exp (X\beta )$, on the estimated parameter values $\hat{\beta }$, and on tests for overdispersion are presented in Additional file 1.

To estimate the number of cases on day *d* without vaccination, we use the formula: $\sum\nolimits_{a,s,AS} {population_{a,s,AS} \times r_{a,s,d,AS} }$.

To estimate the number of cases on day *d* with vaccination, we use: $\sum\nolimits_{a,s,AS} {population_{a,s,AS} \times r_{a,s,d,AS} } \left( {1 - \rho_{1} p_{1,a,s,d,AS} - \rho_{2} p_{2,a,s,d,AS} } \right)$.

Summing over *d* gives the total numbers of cases without and with vaccination, the difference of which is the total number of cases averted. By plotting the numbers of cases without and with vaccination on day *d* against *d*, the numbers of cases averted can be visualized as the area between these curves.

Confidence intervals are based on asymptotic normal theory and are two-sided with level 0.05, except when an effectiveness estimate equals one, in which case a one-sided level 0.05 lower confidence limit is used (pp. 224–227, [11]).

## Results

### Vaccinations reported over time

**Fig. 1:**
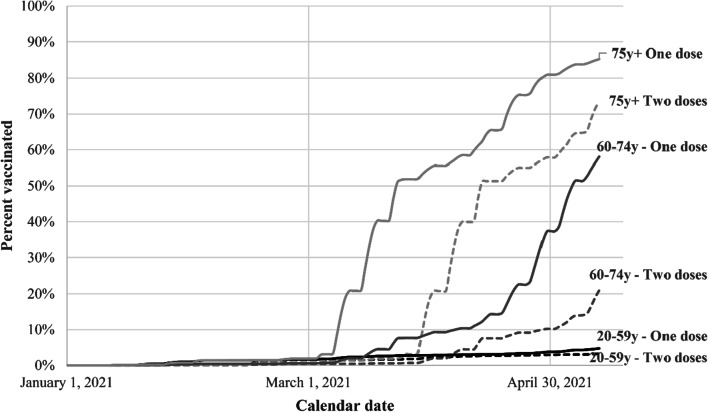
Percentage of people vaccinated with 1 and with 2 doses by age group plotted against calendar time (January 1, 2021 to May 13, 2021). A person is defined as vaccinated with two doses on a given day if the second dose was received at least 14 days previously. A person is defined as vaccinated with one dose on a given day if the first vaccination was received at least 14 days previously and no second dose was given more than 14 days previously. Black, dark gray and light gray lines correspond respectively to 20–59, 60–74 and ≥ 75-year age groups. Solid lines are for one dose and dashed lines for two doses

There was little vaccination before early March 2021 (Fig. 1). Receipt of the first dose accelerated in the first half of March for those aged 75 or older and later in March and early April for those aged 60–74. By May 13, 2021, 85% of those aged 75 or more had received one dose at least 14 days earlier and 73% had received two doses at least 14 days earlier. Among those aged 60–74, 58% had received one dose and 21% two doses. Only 5% of those aged 20–59 had received one dose.

### Incidence of COVID-19 cases, hospitalizations and deaths

From December 1, 2020 to May 13, 2021, 125,031 COVID-19 cases were diagnosed, representing 3.5% of the population aged 20 and above (Table 1). The percentages diagnosed with COVID-19 exceeded 3% in all age groups between 20 and 59 years and was less than 2% for those over ≥ 75 years old. Of the cases diagnosed, 7716 (6.2%) were hospitalized and 1929 (1.5%) died. The proportion hospitalized increased from 1.3% in those aged 20–24 years to 42.5% of those aged 85 years and above. The proportion dying likewise increased from 0.1% in those aged 20–24 years to 27.3% in those aged 85 years and above.

**Table 1:** Estimated population size, number of COVID-19 cases, number of cases who were hospitalized, and number of cases who died between December 1, 2020 and May 13, 2021, by age-group

Age-class	Population (in thousands)	Cases (and percent of the population)	Hospitalizations (and percent of the cases)	Deaths (and percent of the cases)
20–24	416	13,747 (3.3%)	177 (1.3%)	9 (0.1%)
25–29	437	16,928 (3.9%)	271 (1.6%)	11 (0.1%)
30–34	452	17,928 (4.0%)	400 (2.2%)	25 (0.1%)
35–39	427	16,164 (3.8%)	495 (3.1%)	36 (0.2%)
40–44	356	13,679 (3.8%)	545 (4.0%)	64 (0.5%)
45–49	284	11,022 (3.9%)	612 (5.6%)	87 (0.8%)
50–54	274	10,255 (3.7%)	785 (7.7%)	113 (1.1%)
55–59	267	9,191 (3.4%)	1010 (11.0%)	206 (2.2%)
60–64	227	6,264 (2.8%)	909 (14.5%)	241 (3.8%)
65–69	174	4,058 (2.3%)	765 (18.9%)	277 (6.8%)
70–74	118	2,375 (2.0%)	564 (23.7%)	210 (8.8%)
75–79	82	1,509 (1.8%)	440 (29.2%)	209 (13.9%)
80–84	55	968 (1.8%)	343 (35.4%)	184 (19.0%)
85 +	52	943 (1.8%)	400 (42.4%)	257 (27.3%)
Total	3621	125,031 (3.5%)	7716 (6.2%)	1929 (1.5%)

**Fig. 2:**
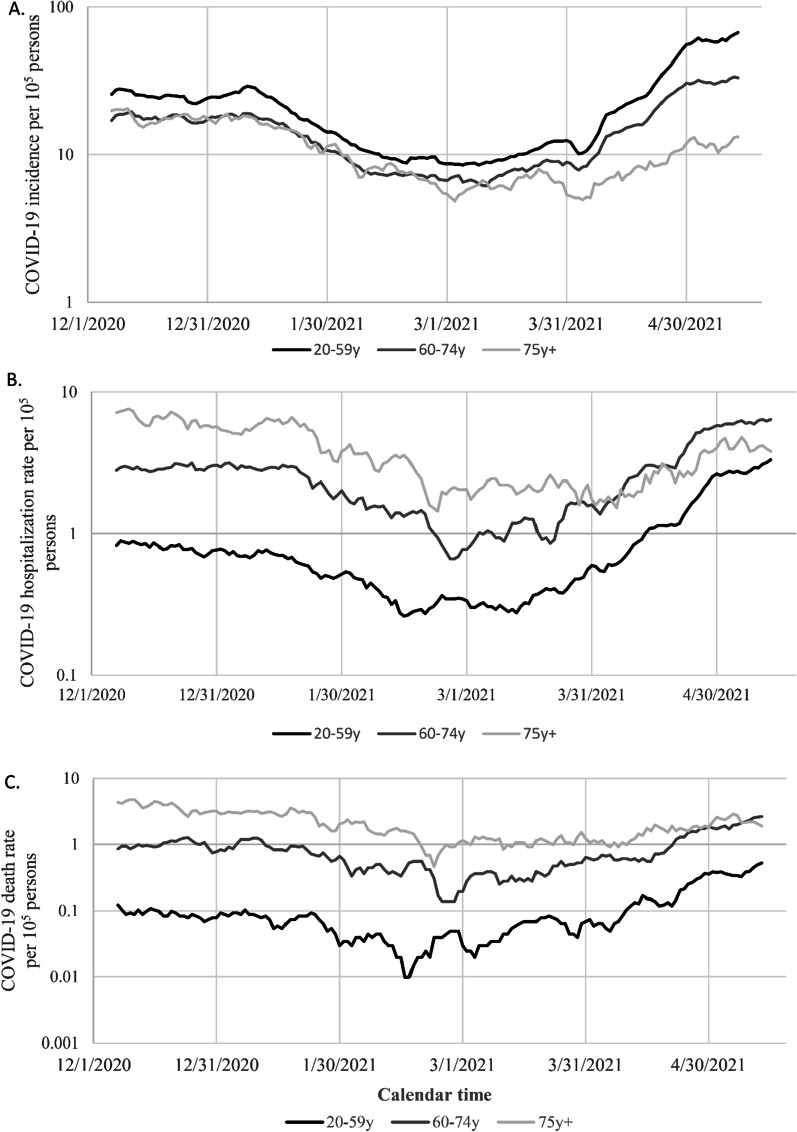
Semi-logarithmic plot of COVID-19 incidence (**A**), hospitalizations (**B**), and deaths (**C**) per 10^5^ people against calendar time (December 7, 2020 to May 13, 2021) by age group. Incidence, hospitalizations, and deaths were estimated from the preceding seven-day moving average. Black, dark grey and light grey lines correspond respectively to 20–59, 60–74 and ≥ 75-year age groups

The daily seven-day average COVID-19 incidence rate per 10^5^ is plotted on a logarithmic scale against day of the epidemic, separately for those aged 20–59, 60–74, and ≥ 75 years (Fig. 2A). The incidence profiles for the three age groups are similar but vertically displaced until late March 2021, when appreciable vaccination had occurred in the ≥ 75 age group (see Fig. 1). Beginning in late March, the slope of the curve for the ≥ 75 age group became substantially less than for the other age groups, reflecting slower exponential growth, and the slope for the 60–74 year age group became less than that of the 20–59 year age group, whose members had received little vaccine.

These patterns were even more pronounced for the incidence rates of COVID-19-related hospitalizations (Fig. 2B) and deaths (Fig. 2C).

Before appreciable vaccination (December 1, 2020 to March 8, 2021), people aged 20–59 years old accounted for 18.3% of the deaths; from March 9 to May 13, 2021, they had received few vaccinations and accounted for 39.5% of the deaths.

### Estimated vaccine effectiveness

Estimates of vaccine effectiveness against COVID-19 incidence (${\rho }_{1}$ and ${\rho }_{2}$ in Eq. (1)) were 59% (95% confidence interval 53% to 64%) for one dose and 93% (90% to 96%) for two doses (rows 2 and 3 of Table 2). For preventing hospitalizations, the estimated efficacies were 76% (68% to 85%) for one dose and 100% (97% to 100%) for two doses. For deaths, the estimated efficacies were 63% (47% to 80%) for one dose and 100% (97% to 100%) for two doses.

**Table 2:** Estimated effectiveness of one and two doses of vaccination (all or primarily tozinameran) to prevent diagnosed cases, hospitalized cases and deaths from the present ecologic study in Costa Rica and from other selected studies

References	Location	Dates covered	Doses	Comments	Incident cases	Hospitalizations	Deaths
Current study	Costa Rica	12/1/2020–5/13/2021	1	Ecologic study design. Primarily Alpha (B1.1.7) variant	**59% (53 to 64)**^**a**^	**76% (68 to 85)**	**63% (47 to 80)**
			2		**93% (90 to 96)**^**a**^	**100% (97 to 100)**	**100% (97 to 100)**
Haas et al. [3]	Israel	1/24/2021–4/3/2021	2	Observational cohort study. Primarily Alpha variant	97∙0% (96∙7 to 97∙2)^b^	97∙2% (96.8 to 97.5)	96∙7% (96.0 to 97.3)
Chung et al. [6]	Ontario, Canada	12/14/2020–4/19/2021	1	Community-based test-negative case–control design. Alpha variant primarily	59% (55 to 62)^b^	NA	69% (59–77)^f^
			2		91% (88 to 93)^b^	NA	96% (82–99)^f^
Polack et al. [2]	Multinational, primarily US	7/27/2020–11/14/2020	2	Randomised, double-blinded trial. Probably the Alpha variant	94∙6% (89.9 to 97.3)^b^	NA	NA
Chemaitelly et al. [5]	Qatar	1/1/2021–9/5/2021	1	Test-negative case–control design. Beta and Delta variants	36∙8% (33.2 to 40.2)^c^	NA	NA
			2		77∙5% (76.4 to 78.6)^c^	NA	NA
Tang et al. [4]	Qatar	3/23/2021–9/7/2021	1	Test-negative case–control design against Delta variant	45.3% (22.0 to 61.6)^c^	NA	79∙7% (− 59.5 to 97.4)^d^
			2		51.9% (47.0 to 56.4)^c^	NA	93∙4% (85.4 to 97.0)^d^
Tenforde et al. [7]	United States	3/11/2021–8/15/2021	2	Hospital-based case–control. Alpha and Delta variants	NA	85% (82 to 87)	NA^e^

*NA* not available

^a^Symptomatic or exposed to infected family member

^b^Symptomatic

^c^Symptomatic and non-symptomatic

^d^Severe, critical or fatal outcome

^e^Data not presented separately for tozinameran

^f^Death or hospitalization

### Estimated numbers of incident cases, hospitalizations and deaths averted by vaccination

**Fig. 3:**
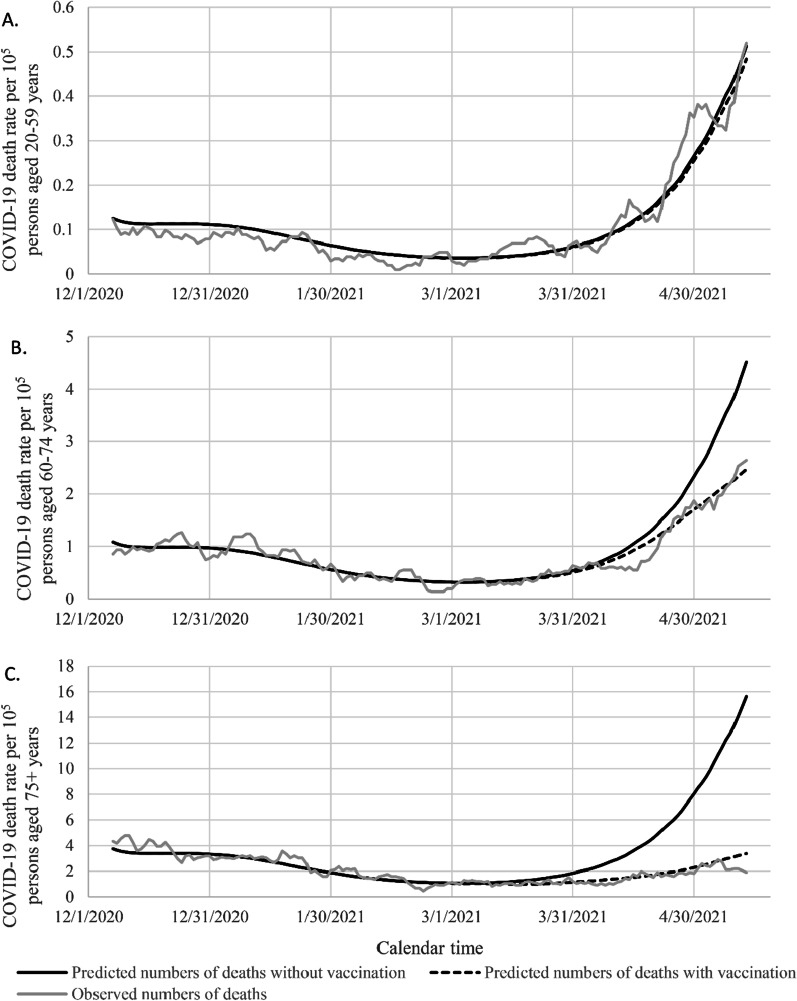
Plots of predicted numbers of COVID-19 deaths against day of epidemic without vaccination (black solid line) and with vaccination (black dashed line) respectively for persons aged 20–59 (**A**), 60–74 (**B**), and 75 + (**C**) years. The area between these plots represents the estimated number of deaths prevented by vaccination. Also shown are the observed numbers of deaths (seven-day average, grey line), which agree well with the dashed line that accounts for vaccination

Figure 3A shows the predicted number of deaths absent vaccination (solid line) and with vaccination (dashed line) for persons aged 20–59 years during the study period.

The area between these curves is the number of deaths averted. The variance of the estimated number of cases averted is computed as in Additional file 1. The jagged line represents the seven-day average observed numbers of deaths and follows the dashed line that accounts for vaccination. We estimated that only 15 (13 to 19) deaths were averted in those aged 20–59 years. Figure 3B and C present analogous data for those aged 60–74 years and 75 + years. For those aged 60–74 years, we estimated 155 (128 to 202) deaths averted and for those aged 75 + years we estimated 451 (410 to 535) deaths averted. The youngest group, that received little vaccine, shows little departure of the observed deaths from those predicted absent vaccine (solid line), but the older groups, that received vaccine, show an important divergence between the observed deaths and those predicted to have occurred absent vaccination. Overall, we estimated that 621 (556 to 750) deaths were averted (see Additional file 1: Fig. S1).

A similar analysis of hospitalizations (Additional file 1: Figs. S2, S3, S4, S5) shows 112 (108 to 119) hospitalizations averted for those aged 20–59, 481 (432 to 547) hospitalizations averted for those aged 60–74 years, and 869 (797 to 945) hospitalizations averted for those aged 75 + years. An analysis of COVID-19 incidence (Additional file 1: Figs. S6, S7, S8, S9) shows 1938 (1878 to 2006) infections averted for those aged 20–59 years, 1559 (1449 to 1664) infections averted for those aged 60–74 years, and 1643 (1558 to 1758) infections averted for those aged 75 + years.

### Sensitivity analyses

We conducted analyses to assess the sensitivity of estimates of vaccine effectiveness to certain analytic choices used in the main analysis (see Additional file 1). Our results were not materially changed by: (1) setting the proportion with one dose in an ecologic unit equal to the maximum of the observed proportion with one dose and the observed proportion with two doses; (2) reducing population sizes in other groups proportionally to preserve census totals when the population size in some age × gender × *AS* group is increased to the maximum of the census estimate and the number of observed vaccinees; and (3) for two doses, changing the starting date of the analysis from December 1, 2020 to February 1, 2021. However, for one dose, this change in starting date reduced the estimates of effectiveness somewhat compared to Table 2, possibly because few hospitalizations and deaths occurred between first and second doses, resulting in large confidence intervals for estimates of the effectiveness of one dose (Table 2 and Additional file 1).

## Discussion

We developed a novel Poisson regression model for estimating vaccine effectiveness from ecologic data. We used these methods to estimate the effectiveness of vaccination in the general population of Costa Rica aged 20 years and older. This approach can be applied to regions where ecologic data on disease incidence and vaccinations are available, but individual-level data are not. In Costa Rica, at a time before the Delta variant had appeared and practically all vaccinations were with tozinameran, this approach yielded vaccine effectiveness point estimates for one dose of 59% for incidence of COVID-19 (PCR confirmed or symptomatic and exposed to an infected household member), 76% for hospitalizations, and 63% for death. For two doses, the estimates of effectiveness were 93% for incident COVID-19, and 100% for hospitalizations and deaths.

These ecologic associations are consistent with previous literature on the effectiveness of tozinameran against the Alpha variant from observational studies and a randomized trial (rows 4 to 7 in Table 2). For one dose, the Canadian study [6] is most relevant and gives almost identical results as we found for incidence and death (Table 2). For two doses, our estimates for incident COVID-19 are consistent with the findings in Israel [3], Canada [6], and with the randomized trial in the US and elsewhere [2], in view of the overlapping confidence intervals. Our confidence intervals for effectiveness against hospitalizations and deaths are also compatible with the corresponding data from Israel [3] and Canada [6]. The other studies in Table 2 included patients with the Delta variant for which tozinameran had lower efficacies (rows 8 to 12 in Table 2). A pre-print of a recent case–control study in Costa Rica reported 93% effectiveness against hospitalizations [12]. However, it included a period in which both tozinameran and the Oxford/AstraZeneca vaccines were used, and the Delta variant was probably dominant.

We were surprised that the ecologic associations were in such good agreement with those from individual-level data, because ecologic associations can be confounded by unmeasured individual-level covariates [13]. In Costa Rica, however, where there is universal health coverage [14] and good acceptance of vaccination programs, confounding effects related to differential access to vaccinations and other protective behaviors across geographic units might be small. In countries where better educated or wealthier individuals tend to have better access to vaccination and also tend to take protective measures like wearing a mask or working from home, studies that do not adjust for these protective measures, such as ecologic studies and even individual-level studies without data on protective measures, can produce upwardly biased estimates of vaccine effectiveness that reflect the effects of the protective measures in addition to the effects of vaccination. Further comparisons of ecologic vaccine effectiveness estimates against estimates from individual level studies are needed to check the validity of the ecologic approach in various settings.

Estimates of effectiveness can reflect both the direct effects of vaccination on the immune system of a vaccinee and indirect effects that result from reduced exposure (“force of infection”) of the vaccinee from other members of the community who have been vaccinated [15], a phenomenon that is sometimes called “herd immunity.” Such indirect effects are present not only in ecologic studies but also in individual-level studies of vaccine effectiveness. In randomized individual-level vaccine trials, exposure to infection, even in the presence of herd immunity, is similar in vaccinated and unvaccinated subjects, and estimates of vaccine efficacy are unbiased. In observational individual-level studies in which vaccinated and unvaccinated subjects come from the same regions, there is little or no bias from herd immunity, but bias can be appreciable if they come from different regions with different levels of herd immunity. In ecologic studies the potential for bias is greater because regions with high proportions vaccinated will tend to have more herd immunity than regions with low proportions vaccinated (although some herd immunity may also be conferred by previous infection, not vaccination).

In addition to permitting estimation of vaccine effectiveness, our model allows one to estimate the numbers of deaths, hospitalizations and incident infections that were prevented by vaccination. Such data quantify the public health benefits of vaccination. An alternative approach for estimating the numbers of incident cases, hospitalizations, and deaths prevented by vaccination is to fit a complex compartmental model to the data on incidence, hospitalizations, and deaths and to use estimates of vaccine efficacy taken from other studies [16]. This approach requires assumptions on mixing of subpopulations, transmissibility of the virus, and other parameters that are not required by our method, and unlike our method, does not yield an estimate of vaccine effectiveness.

This study has additional limitations apart from the ecologic design. As described in the Methods, the populations of the Áreas de Salud were estimated by combining data from the Tribunal Supremo de Elecciones with census data to account for non-native inhabitants. In some ecologic units the numbers of hospitalizations and deaths were small or zero. Modeling the rate for unvaccinated persons in all the ecologic units, corresponding to the factor $\exp (X\beta )$ in Eq. (1), was done carefully, but there is no unique way to create such a model. Unreported calculations showed that the conclusions on vaccine effectiveness were robust to variations in such models. We have assumed that vaccine effectiveness is constant over ecologic units, including age categories, in Eq. (1). This assumption is supported by data from Israel showing nearly constant effectiveness against infections, hospitalizations and deaths across age groups [3]. Because we had very few deaths in younger age groups, we were not able to estimate age-group-specific vaccine effectiveness reliably. The homogeneous vaccination effectiveness model fits the observed data well for all three age groups (Fig. 3A–C), even though most of the data come from older age groups (Table 1). Thus, heterogeneity in vaccine effectiveness across age groups, if present, does not reflect itself in failures of the homogeneous model to describe incidence trends. We used data on hospitalizations and deaths through July 5, 2021, which is nearly two months after the end of the study period. Thus, we have ascertained the vast majority of hospitalizations and deaths that followed a diagnosis of COVID-19 during the study. Our vaccine effectiveness therefore pertains to the incidence of COVID-19 diagnoses that later result in hospitalization or death. Our ecologic data do not include individual dates of death. If we could use dates of death, vaccine effectiveness would refer to deaths among persons with a previous COVID-19 diagnosis. It is not clear that this definition of effectiveness is preferable.

Regarding the substantive conclusions, our data reflect tozinameran vaccination almost exclusively and during a period before the Delta variant was present. Moreover, the vaccine data pertained mainly to those aged 60 and over, because little vaccine was given to the younger age groups.

During our study, only 3.5% of the population became infected. One could adapt these methods for monitoring vaccine effectiveness over calendar time, while taking immunity from previous infections into account, if ecologic data on the joint prevalence of previous COVID-19 infection and vaccination are available. To account for multiple variants, the surveillance system would need to report variant-specific incidence within ecologic units, which would require large-scale genotyping. Without data on variant-specific incidence, the ecologic approach could estimate effectiveness against the concurrent mixture of variants, which is of public health importance.

In summary this novel ecologic analysis yielded vaccine effectiveness results in Costa Rica that were consistent with findings based on individual-level data in other countries where tozinameran was evaluated against the Alpha variant. This ecologic approach may be useful in other areas where individual-level studies are not feasible. It may also be adapted for monitoring effectiveness over calendar time.

## Supplementary Information

**Additional file 1: Figures S1-S9.** Statistical Methods and Model Details, Including Supplementary Table S1 and Sensitivity Analyses

**Additional file 2.** Other members of the RESPIRA Study Group

## Data Availability

Data for COVID-19 incidence, hospitalizations and deaths can be found at: http://geovision.uned.ac.cr/oges/evolucioncovid.html. Data for vaccination is not available online. We obtained it by applying to the Caja Costarricense de Seguro Social, Costa Rica. Please contact Romain Fantin (ROMAIN.FANTIN@ucr.ac.cr) if you have questions concerning how to obtain these data.

## Abbreviations

AS: Áreas de Salud or health areas
CCSS: Caja Costarricense del Seguro Social
COVID-19: Novel coronavirus disease
mRNA: Messenger ribonucleic acid
PCR: Polymerase chain reaction
SARS-CoV-2: Severe acute respiratory syndrome coronavirus 2
