# Supplementary material for: Estimating vaccine effectiveness against SARS-CoV-2 infection, hospitalization and death from ecologic data in Costa Rica
Source: BMC Infect Dis. 2022 Oct 2;22:767. doi: 10.1186/s12879-022-07740-5 (PMC9526815; doi:10.1186/s12879-022-07740-5)
Supplement: Supplementary file 1 — Additional file 1: Figures S1-S9. Statistical Methods and Model Details, Including Supplementary Table S1 and Sensitivity Analyses [file 12879_2022_7740_MOESM1_ESM.docx]

**Additional File 1**

**Estimating vaccine effectiveness against SARS-CoV-2 infection, hospitalization and death from ecologic data in Costa Rica**

Romain Fantin^1,2,*^, Rolando Herrero^2^, Allan Hildesheim^3^, Cristina Barboza-Solís^4^, Amada Aparicio^5^, Rebecca Prevots^6^, Ruth M Pfeiffer^7^, Mitchell H Gail^7,*^ for the RESPIRA Study Group

**^1^Centro Centroamericano de Población , Universidad de Costa Rica
^2^Agencia Costarricense de Investigaciones Biomédicas, Fundación INCIENSA, Costa Rica
^3^Independent**^4^**Facultad de Odontología, Universidad de Costa Rica**^5^**Centro de Desarrollo Estratégico e Información en Salud y Seguridad Social (CENDEISSS) Caja Costarricense de Seguro Social, Costa Rica**^6^**Epidemiology and Population Studies Unit, Division of Intramural Research, National Institute of Allergy and Infectious Diseases, USA
^7^Biostatistics Branch, Division of Epidemiology and Genetics, National Cancer Institute, USA**

*Correspondence to: Romain Fantin, Centro Centroamericano de Población, Universidad de Costa Rica San José 2060, Costa Rica **ROMAIN.FANTIN@ucr.ac.cr** and Mitchell H Gail, Division of Cancer Epidemiology and Genetics, National Cancer Institute, 9609 Medical Center Drive RM 7-E138, MSC 9780, Bethesda MD 20892, USA **gailm@mail.nih.gov**

**Table of Contents:**

Figures S1-S9
Statistical Methods and Model Details, Including Supplementary Table S1 and Sensitivity Analyses

Supplementary Figures

**
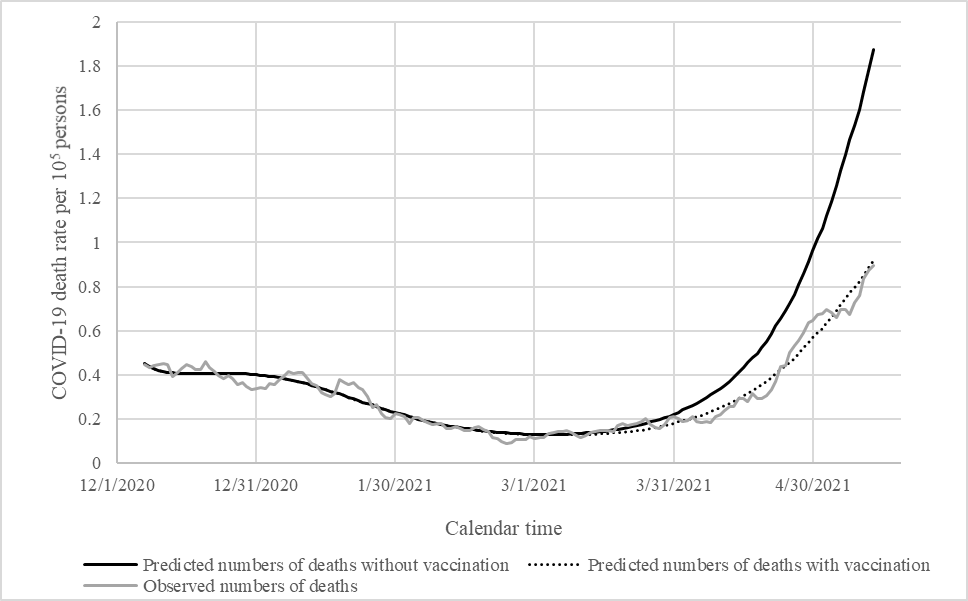
**

**Supplementary Figure S1.** Plots of predicted numbers of Covid-19 deaths against day of epidemic without vaccination (black solid line) and with vaccination (black dotted line). The area between these plots represents the estimated number of deaths prevented by vaccination. Also shown are the observed numbers of deaths (seven-day average, grey solid line), which agree well with the dotted line that accounts for vaccination. This plot is for all persons aged 20 years and older.

**Supplementary Figure S2.** Plot of predicted numbers of Covid-19 hospitalizations against day of epidemic without vaccination (black solid line) and with vaccination (black dotted line). The area between these plots represents the estimated number of hospitalizations prevented by vaccination. Also shown are the observed numbers of hospitalizations (seven-day average, grey solid line), which agree well with the dotted line that accounts for vaccination. This plot is for all persons aged 20 years and older.

**Supplementary Figure S3.** Plot of predicted numbers of Covid-19 hospitalizations against day of epidemic without vaccination (black solid line) and with vaccination (black dashed line) for persons aged 20-59 years. Also shown are the observed numbers of hospitalizations (seven-day average, grey solid line).

**Supplementary Figure S4.** Plot of predicted numbers of Covid-19 hospitalizations against day of epidemic without vaccination (black solid line) and with vaccination (black dashed line) for persons aged 60-74 years. Also shown are the observed numbers of hospitalizations (seven-day average, grey solid line).

**Supplementary Figure S5.** Plot of predicted numbers of Covid-19 hospitalizations against day of epidemic without vaccination (black solid line) and with vaccination (black dashed line) for persons aged 75+ years. Also shown are the observed numbers of hospitalizations (seven-day average, grey solid line).

**Supplementary Figure S6.** Plot of predicted numbers of Covid-19 incident cases against day of epidemic without vaccination (black solid line) and with vaccination (black dotted line). The area between these plots represents the estimated number of cases prevented by vaccination. Also shown are the observed numbers of cases (seven-day average, grey solid line), which agree well with the dotted line that accounts for vaccination. This plot is for all persons aged 20 years and older.

**Supplementary Figure S7.** Plot of predicted numbers of Covid-19 incident cases against day of epidemic without vaccination (black solid line) and with vaccination (black dashed line) for persons aged 20-59 years. Also shown are the observed numbers of cases (seven-day average, grey solid line), which agree well with the dashed line that accounts for vaccination.

**Supplementary Figure S8.** Plot of predicted numbers of Covid-19 incident cases against day of epidemic without vaccination (black solid line) and with vaccination (black dashed line) for persons aged 60-74 years. Also shown are the observed numbers of cases (seven-day average, grey solid line), which agree well with the dashed line that accounts for vaccination.

**Supplementary Figure S9.** Plot of predicted numbers of Covid-19 incident cases against day of epidemic without vaccination (black solid line) and with vaccination (black dashed line) for persons aged 75+ years. Also shown are the observed numbers of cases (seven-day average, grey solid line), which agree well with the dashed line that accounts for vaccination.

**Statistical Methods and Model Details, Including Supplementary Table S1 and Sensitivity Analyses**

**Derivation of equation (1) in the paper for the expected numbers of events**

As in the paper, let$a,s,d, \text{and} AS$ represent the age group, sex, day of epidemic and *Áreas de Salud* respectively. The expected numbers of events in the ecologic unit $(a,s,d,AS)$ is $E(Y_{a,s,d,AS})=population_{a,s,AS}\times\exp(X\beta)\times(1-\rho_{1}p_{1,a,s,d,AS}-\rho_{2}p_{2,a,s,d,AS})$, where $\exp(X\beta)\equiv r_{a,s,d,AS}$represents the incidence rate in the absence of vaccination in the ecologic unit $(a,s,d,AS)$ (see below for details), $population_{a,s,AS}$is the population size, assumed to be constant over days $d$, and $\rho_{1}$and$\rho_{2}$ are the vaccine efficacies for one and two doses respectively. The quantities $p_{1,a,s,d,AS}$ and $p_{2,a,s,d,AS}$ are respectively the proportions of the ecologic unit who have had only one vaccination at least 14 days before day *d* and who have had two vaccinations at least 14 days before day *d,* and $p_{0,a,s,d,AS}=1-p_{1,a,s,d,AS}-p_{2,a,s,d,AS}.$ If the relative risks of an event compared to unvaccinated persons are $rr_{1}$ for one dose and $rr_{2}$ for two doses, then the expected number of events is

$$\begin{matrix} E(Y_{a,s,d,AS})=population_{a,s,AS}\times\exp(X\beta)\times(p_{0,a,s,d,AS}+rr_{1}p_{1,a,s,d,AS}+rr_{2}p_{2,a,s,d,AS}) \\ =population_{a,s,AS}\times\exp(X\beta)\times\{1-(1-rr_{1})p_{1,a,s,d,AS}-(1-rr_{2})p_{2,a,s,d,AS}\} \\ \equiv population_{a,s,AS}\times\exp(X\beta)\times(1-\rho_{1}p_{1,a,s,d,AS}-\rho_{2}p_{2,a,s,d,AS}). \end{matrix}$$

**Modeling the incidence rate in the absence of vaccination,** $\exp(X\beta)\equiv r_{a,s,d,AS}$

A saturated model would have a separate parameter for each ecologic unit and would fit the data perfectly but not allow for estimation of vaccine effectiveness. The number of parameters would be 14 age groups x 2 sexes x 105 *AS* x 164 days = 482,160. Instead, we considered seven exclusive and exhaustive regions of Costa Rica for time trends. Let $region(AS)$be the region into which *Áreas de Salud* (*AS*) falls. We allow for different time trends in each region via the model.

Thus, each *AS* except number 105 has its own parameter $\tau_{AS}$ that adds to an overall intercept parameter, $\mu$. Each region has its own coefficients in the sixth-degree polynomial for time trend measured in days *d* beginning at day *d*=0 on December 1, 2020. The parameters ω_weekday_ represent the day of the week effect with ω_SUNDAY_=0 for identifiability. The parameters $\alpha_{a}$represent age effects with$\alpha_{20-24}=0$for identifiability. The parameters $\gamma_{s}$represent gender effects with $\gamma_{female}=0$for identifiability. The expectation model has 1 intercept +104+6x7+13+1+6=167 $\beta$parameters plus the two effectiveness parameters. Estimates of these 169 parameters are given in supplemental Table S1 together with their 95% confidence intervals, separately for Covid-19 incidence, hospitalizations, and deaths.

Alternative models taking into account the cumulative incidence of COVID-19 infections in each AS since the beginning of the pandemic have been tested. The results were similar to the results presented in this study.

**Supplementary Table S1. Estimates of the parameters for the Covid-19 incidence, Covid-19 hospitalizations, Covid-19 deaths models, with their 95% confidence intervals.**

| **Variables** | **Parameter estimates  [95%CI] Incidence model** | **Parameter estimates [95%CI] Hospitalizations** | **Parameter estimates [95%CI] Deaths** |
| --- | --- | --- | --- |
| AS 1 | -0∙05 [-0∙24 – 0∙14] | 0∙49 [-0∙15 – 1∙12] | 1∙20 [0∙13 – 2∙27] |
| AS 2 | -0∙55 [-0∙67 – -0∙43] | -1∙02 [-1∙52 – -0∙51] | -1∙28 [-2∙3 – -0∙25] |
| AS 3 | 0∙04 [-0∙14 – 0∙21] | -0∙25 [-0∙97 – 0∙48] | 0∙11 [-1∙17 – 1∙39] |
| AS 4 | 0∙19 [0∙08 – 0∙30] | 0∙05 [-0∙36 – 0∙46] | 0∙41 [-0∙32 – 1∙15] |
| AS 5 | 0∙09 [-0∙02 – 0∙20] | 0∙02 [-0∙39 – 0∙43] | 0∙17 [-0∙57 – 0∙91] |
| AS 6 | 0∙10 [-0∙01 – 0∙21] | 0∙01 [-0∙41 – 0∙43] | 0∙50 [-0∙25 – 1∙25] |
| AS 7 | -0∙08 [-0∙19 – 0∙03] | -0∙12 [-0∙55 – 0∙31] | 0∙45 [-0∙32 – 1∙22] |
| AS 8 | -0∙02 [-0∙09 – 0∙05] | -0∙29 [-0∙55 – -0∙04] | -0∙12 [-0∙55 – 0∙32] |
| AS 9 | 0∙14 [-0∙00 – 0∙28] | 0∙62 [0∙15 – 1∙10] | 1∙08 [0∙20 – 1∙97] |
| AS 10 | -0∙47 [-0∙55 – -0∙40] | -0∙84 [-1∙13 – -0∙55] | -0∙94 [-1∙47 – -0∙40] |
| AS 11 | -0∙07 [-0∙20 – 0∙05] | -0∙45 [-0∙95 – 0∙05] | -0∙35 [-1∙30 – 0∙59] |
| AS 12 | -0∙22 [-0∙41 – -0∙03] | 0∙41 [-0∙21 – 1∙03] | -0∙86 [-2∙93 – 1∙21] |
| AS 13 | 0∙12 [-0∙04 – 0∙29] | -0∙41 [-1∙15 – 0∙34] | -1∙08 [-2∙7 – 0∙54] |
| AS 14 | -0∙04 [-0∙16 – 0∙08] | -0∙01 [-0∙46 – 0∙43] | 0∙15 [-0∙68 – 0∙97] |
| AS 15 | 0∙13 [0∙02 – 0∙25] | -0∙20 [-0∙65 – 0∙25] | 0∙09 [-0∙73 – 0∙91] |
| AS 16 | -0∙12 [-0∙31 – 0∙06] | 1∙01 [0∙51 – 1∙52] | 1∙49 [0∙57 – 2∙41] |
| AS 17 | 0∙20 [0∙05 – 0∙34] | -0∙16 [-0∙82 – 0∙50] | 0∙53 [-0∙66 – 1∙72] |
| AS 18 | -0∙33 [-0∙46 – -0∙21] | 0∙25 [-0∙20 – 0∙69] | 0∙04 [-0∙87 – 0∙95] |
| AS 19 | 0∙43 [0∙28 – 0∙58] | 1∙34 [0∙85 – 1∙83] | 1∙44 [0∙48 – 2∙40] |
| AS 20 | -0∙03 [-0∙10 – 0∙03] | -0∙71 [-0∙95 – -0∙47] | -1∙03 [-1∙48 – -0∙59] |
| AS 21 | -0∙17 [-0∙24 – -0∙10] | -0∙60 [-0∙85 – -0∙35] | -0∙74 [-1∙18 – -0∙29] |
| AS 22 | -0∙11 [-0∙28 – 0∙06] | 0∙23 [-0∙38 – 0∙84] | 0∙79 [-0∙32 – 1∙90] |
| AS 23 | -0∙09 [-0∙26 – 0∙09] | -0∙85 [-1∙63 – -0∙06] | -2∙17 [-4∙31 – -0∙03] |
| AS 24 | -0∙19 [-0∙39 – -0∙00] | -0∙78 [-1∙61 – 0∙05] | -1∙58 [-3∙42 – 0∙26] |
| AS 25 | 0∙46 [0∙30 – 0∙63] | 0∙43 [-0∙24 – 1∙10] | 0∙37 [-0∙95 – 1∙68] |
| AS 26 | -0∙24 [-0∙56 – 0∙07] | 0∙07 [-1∙14 – 1∙28] | -13∙73 [-14∙53 – -12∙93] |
| AS 27 | -0∙22 [-0∙29 – -0∙15] | -0∙38 [-0∙62 – -0∙15] | -0∙75 [-1∙19 – -0∙30] |
| AS 28 | -0∙86 [-1.00– -0∙72] | -1∙70 [-2∙40 – -0∙99] | -1∙18 [-2∙20 – -0∙15] |
| AS 29 | 0∙45 [0∙27 – 0∙63] | 1∙02 [0∙54 – 1∙50] | 1∙48 [0∙63 – 2∙34] |
| AS 30 | -0∙23 [-0∙41 – -0∙04] | -0∙30 [-0∙86 – 0∙27] | -0∙67 [-1∙89 – 0∙55] |
| AS 31 | -0∙08 [-0∙15– -0∙01] | -0∙33 [-0∙58 – -0∙09] | -0∙31 [-0∙74 – 0∙12] |
| AS 32 | 1∙59 [1∙43 – 1∙75] | 0∙17 [-0∙63 – 0∙98] | -0∙04 [-1∙68 – 1∙59] |
| AS 33 | -0∙36 [-0∙44– -0∙29] | -0∙46 [-0∙73 – -0∙20] | -0∙70 [-1∙19 – -0∙20] |
| AS 34 | -0∙16 [-0∙24– -0∙09] | -0∙64 [-0∙91 – -0∙38] | -0∙59 [-1∙07 – -0∙12] |
| AS 35 | -0∙39 [-0∙47– -0∙30] | -0∙50 [-0∙81 – -0∙20] | -0∙23 [-0∙74 – 0∙29] |
| AS 36 | 0∙13 [0∙06 – 0∙20] | -0∙79 [-1∙09 – -0∙50] | -1∙33 [-1∙98 – -0∙68] |
| AS 37 | -0∙26 [-0∙33– -0∙18] | -0∙65 [-0∙91 – -0∙38] | -1∙18 [-1∙73 – -0∙63] |
| AS 38 | 0∙39 [0∙23 – 0∙55] | -0∙46 [-1∙22 – 0∙30] | -1∙12 [-2∙70 – 0∙46] |
| AS 39 | 0∙29 [0∙11 – 0∙47] | 0∙27 [-0∙44 – 0∙97] | 0∙28 [-1∙05 – 1∙61] |
| AS 40 | 0∙36 [0∙18 – 0∙53] | 0∙26 [-0∙44 – 0∙95] | 0∙40 [-0∙91 – 1∙71] |
| AS 41 | 0∙79 [0∙63 – 0∙95] | -0∙27 [-1∙05 – 0∙51] | -1∙87 [-3∙71 – -0∙04] |
| AS 42 | -0∙17 [-0∙25– -0∙09] | -0∙25 [-0∙54 – 0∙05] | -0∙32 [-0∙88 – 0∙24] |
| AS 43 | -0∙02 [-0∙09 – 0∙05] | -0∙22 [-0∙47 – 0∙02] | -0∙31 [-0∙74 – 0∙12] |
| AS 44 | 0∙17 [-0∙01 – 0∙35] | 0∙68 [0∙18 – 1∙18] | 0∙39 [-0∙60 – 1∙38] |
| AS 45 | 0∙08 [-0∙04 – 0∙19] | -0∙33 [-0∙76 – 0∙10] | 0∙09 [-0∙69 – 0∙88] |
| AS 46 | 0∙34 [0∙15 – 0∙53] | 0∙35 [-0∙36 – 1∙07] | 0∙70 [-0∙61 – 2∙00] |
| AS 47 | 0∙53 [0∙39 – 0∙68] | 0∙02 [-0∙66 – 0∙69] | 0∙47 [-0∙72 – 1∙65] |
| AS 48 | 0∙41 [0∙27 – 0∙55] | 0∙33 [-0∙31 – 0∙98] | 0∙32 [-0∙84 – 1∙47] |
| AS 49 | -0∙11 [-0∙18– -0∙03] | -0∙33 [-0∙59 – -0∙08] | -0∙72 [-1∙22 – -0∙22] |
| AS 50 | 0∙05 [-0∙06 – 0∙16] | 0∙08 [-0∙33 – 0∙48] | 0∙25 [-0∙49 – 1∙00] |
| AS 51 | 0∙03 [-0∙08 – 0∙14] | 0∙26 [-0∙15 – 0∙68] | 0∙46 [-0∙31 – 1∙23] |
| AS 52 | 0∙09 [-0∙13 – 0∙31] | -0∙67 [-1∙82 – 0∙49] | 0∙93 [-0∙43 – 2∙29] |
| AS 53 | -0∙41 [-0∙55– -0∙26] | -0∙30 [-0∙83 – 0∙24] | 0∙24 [-0∙75 – 1∙23] |
| AS 54 | 0∙05 [-0∙13 – 0∙23] | 0∙10 [-0∙62 – 0∙82] | 0∙45 [-0∙89 – 1∙78] |
| AS 55 | -0∙30 [-0∙49– -0∙11] | 0∙41 [-0∙21 – 1∙02] | 0∙75 [-0∙43 – 1∙94] |
| AS 56 | 0∙10 [0∙02 – 0∙18] | -0∙17 [-0∙45 – 0∙11] | -0∙58 [-1∙15 – 0∙00] |
| AS 57 | 0∙40 [0∙25 – 0∙55] | 1∙31 [0∙85 – 1∙78] | 1∙02 [0∙10 – 1∙93] |
| AS 58 | 0∙24 [0∙09 – 0∙38] | -0∙03 [-0∙70 – 0∙63] | 0∙51 [-0∙67 – 1∙69] |
| AS 59 | 0∙00 [-0∙18 – 0∙90] | 0∙39 [-0∙29 – 1∙07] | 0∙68 [-0∙58 – 1∙95] |
| AS 60 | -0∙83 [-0∙94– -0∙73] | -1∙28 [-1∙72 – -0∙83] | -1∙42 [-2∙27 – -0∙56] |
| AS 61 | -0∙11 [-0∙20– -0∙03] | -0∙32 [-0∙61 – -0∙04] | -0∙52 [-1∙03 – -0∙00] |
| AS 62 | 0∙32 [0∙17 – 0∙47] | -0∙50 [-1∙26 – 0∙27] | 0∙48 [-0∙76 – 1∙72] |
| AS 63 | 0∙14 [-0∙04 – 0∙32] | -0∙56 [-1∙39 – 0∙27] | -1∙15 [-2∙93 – 0∙62] |
| AS 64 | -0∙34 [-0∙43– -0∙25] | -1∙28 [-1∙73 – -0∙83] | -1∙09 [-1∙84 – -0∙33] |
| AS 65 | -0∙14 [-0∙21– -0∙06] | -0∙33 [-0∙58 – -0∙07] | -0∙33 [-0∙78 – 0∙12] |
| AS 66 | -0∙08 [-0∙29 – 0∙13] | -0∙41 [-1∙33 – 0∙51] | -0∙51 [-2∙61 – 1∙58] |
| AS 67 | 0∙21 [0∙10 – 0∙33] | -0∙19 [-0∙64 – 0∙27] | 0∙08 [-0∙77 – 0∙94] |
| AS 68 | 0∙39 [0∙25 – 0∙54] | 0∙40 [-0∙10 – 0∙89] | 0∙94 [0∙03 – 1∙84] |
| AS 69 | -0∙17 [-0∙24– -0∙09] | -0∙89 [-1∙19 – -0∙58] | -1∙05 [-1∙64 – -0∙47] |
| AS 70 | 0∙53 [0∙37 – 0∙69] | -0∙34 [-1∙09 – 0∙41] | -1∙08 [-2∙69 – 0∙54] |
| AS 71 | 0∙06 [-0∙12 – 0∙25] | 0∙75 [0∙24 – 1∙25] | 1∙28 [0∙36 – 2∙19] |
| AS 72 | 0∙17 [0∙05 – 0∙28] | -0∙05 [-0∙50 – 0∙39] | -0∙04 [-0∙91 – 0∙83] |
| AS 73 | -0∙45 [-0∙53– -0∙37] | -0∙92 [-1∙22 – -0∙62] | -1∙45 [-2∙11 – -0∙79] |
| AS 74 | 0∙60 [0∙43 – 0∙76] | -0∙33 [-1∙09 – 0∙43] | -0∙44 [-1∙98 – 1∙11] |
| AS 75 | -0∙09 [-0∙17– -0∙02] | -0∙15 [-0∙40 – 0∙09] | -0∙33 [-0∙78 – 0∙12] |
| AS 76 | 0∙22 [-0∙00 – 0∙44] | -1∙05 [-2∙11 – 0∙01] | -2∙10 [-4∙59 – 0∙38] |
| AS 77 | -0∙52 [-0∙71– -0∙32] | -0∙60 [-1∙39 – 0∙20] | -0∙40 [-2∙00 – 1∙20] |
| AS 78 | 0∙09 [-0∙03 – 0∙22] | -0∙12 [-0∙6 – 0∙37] | -0∙07 [-1∙02 – 0∙88] |
| AS 79 | -0∙55 [-0∙69– -0∙41] | -0∙13 [-0∙62 – 0∙36] | 0∙45 [-0∙44 – 1∙34] |
| AS 80 | -0∙82 [-0∙92– -0∙72] | -1∙05 [-1∙42 – -0∙68] | -1∙35 [-2∙07 – -0∙63] |
| AS 81 | 0∙19 [0∙01 – 0∙36] | 1∙02 [0∙56 – 1∙48] | 1∙18 [0∙33 – 2∙03] |
| AS 82 | 0∙88 [0∙72 – 1∙03] | -0∙08 [-0∙83 – 0∙66] | -0∙62 [-2∙21 – 0∙97] |
| AS 83 | -0∙2 [-0∙28– -0∙12] | -0∙23 [-0∙5 – 0∙05] | -0∙39 [-0∙89 – 0∙12] |
| AS 84 | -0∙23 [-0∙32 – -0∙15] | -0∙58 [-0∙91 – -0∙26] | -0∙83 [-1∙46 – -0∙20] |
| AS 85 | 0∙18 [0∙05 – 0∙31] | 0∙13 [-0∙35 – 0∙61] | 0∙34 [-0∙61 – 1∙28] |
| AS 86 | -0∙16 [-0∙24 – -0∙80] | -0∙54 [-0∙83 – -0∙26] | -0∙89 [-1∙5 – -0∙29] |
| AS 87 | 0∙09 [-0∙03 – 0∙22] | 0∙39 [-0∙06 – 0∙84] | 0∙36 [-0∙52 – 1∙23] |
| AS 88 | 0∙32 [0∙12 – 0∙51] | -0∙89 [-1∙78 – -0∙00] | -2∙21 [-4∙22 – -0∙20] |
| AS 89 | -0∙03 [-0∙15 – 0∙09] | -0∙0056 [-0∙44 – 0∙43] | 0∙18 [-0∙64 – 1∙00] |
| AS 90 | -0∙51 [-0∙62 – -0∙39] | -0∙47 [-0∙91 – -0∙04] | -0∙24 [-1∙03 – 0∙56] |
| AS 91 | -0,16 [-0∙24 – -0∙09] | -0∙54 [-0∙82 – -0∙25] | -0∙84 [-1∙4 – -0∙28] |
| AS 92 | 0∙02 [-0∙10 – 0∙14] | 0∙01 [-0∙44 – 0∙46] | 0∙13 [-0∙76 – 1∙01] |
| AS 93 | 0∙74 [0∙60 – 0∙89] | 0∙86 [0∙39 – 1∙33] | 1∙12 [0∙25 – 2∙00] |
| AS 94 | 0∙33 [0∙16 – 0∙51] | 0∙21 [-0∙49 – 0∙90] | 0∙39 [-0∙94 – 1∙71] |
| AS 95 | 0∙26 [0∙14 – 0∙38] | 0∙36 [-0∙07 – 0∙80] | 0∙40 [-0∙41 – 1∙22] |
| AS 96 | 0∙58 [0∙44 – 0∙72] | -0∙09 [-0∙76 – 0∙57] | 0∙74 [-0∙42 – 1∙91] |
| AS 97 | -0∙09 [-0∙25 – 0∙06] | 0∙13 [-0∙57 – 0∙83] | 0∙94 [-0∙26 – 2∙15] |
| AS 98 | -0∙25 [-0∙36 – -0∙13] | -0∙14 [-0∙56 – 0∙28] | 0∙22 [-0∙54 – 0∙99] |
| AS 99 | -0∙29 [-0∙40 – -0∙17] | -0∙01 [-0∙43 – 0∙42] | -0∙08 [-0∙89 – 0∙73] |
| AS 100 | -0∙25 [-0∙43 – -0∙06] | -0∙22 [-0∙90 – 0∙46] | -0∙12 [-1∙46 – 1∙21] |
| AS 101 | -0∙61 [-0∙68 – -0∙53] | -0∙47 [-0∙71 – -0∙23] | -0∙98 [-1∙46 – -0∙5] |
| AS 102 | -0∙04 [-0∙19 – 0∙11] | -0∙15 [-0∙72 – 0∙43] | 0∙64 [-0∙31 – 1∙59] |
| AS 103 | 0∙69 [0∙53 – 0∙85] | 0∙02 [-0∙74 – 0∙77] | 0∙49 [-0∙86 – 1∙83] |
| AS 104 | 0∙29 [0∙16 – 0∙41] | -0∙44 [-1∙01 – 0∙13] | -0∙22 [-1∙33 – 0∙9] |
| Day_Region 1_1 | 0∙01 [-0∙02 – 0∙03] | -0∙02 [-0∙08 – 0∙04] | -0∙11 [-0∙23 – 0∙01] |
| Day_Region 1_2 | 0∙0018 [0∙0004 – 0∙0031] | 0∙0024 [-0∙0012 – 0∙006] | 0∙0084 [0∙0013 – 0∙02] |
| Day_Region 1_3 | -0∙000077 [-0∙0001 – -0∙000046] | -0∙000093 [-0∙0002 – -0∙000007] | -0∙0002 [-0∙0004 – -0∙000073] |
| Day_Region 1_4 | 104∙61 [69∙56 – 139∙66] | 128∙04 [30∙38 – 225∙71] | 310∙91 [109∙05 – 512∙76] |
| Day_Region 1_5 | -58∙17 [-76∙77 – -39∙58] | -74∙22 [-126∙66 – -21∙77] | -173∙47 [-282∙87 – -64∙07] |
| Day_Region 1_6 | 11∙66 [7∙9 – 15∙42] | 15∙52 [4∙82 – 26∙22] | 35∙78 [13∙34 – 58∙22] |
| Day_Region 2_1 | -0∙0045 [-0∙03 – 0∙02] | -0∙04 [-0∙11 – 0∙02] | -0∙12 [-0∙25 – 0∙02] |
| Day_Region 2_2 | -0∙0007 [-0∙002 – 0∙0006] | 0∙0008 [-0∙0034 – 0∙0049] | 0∙0047 [-0∙0038 – 0∙01] |
| Day_Region 2_3 | 0∙000012 [-0∙00002 – 0∙000043] | -0∙000009 [-0∙0001 – 0∙000094] | -0∙0001 [-0∙0003 – 0∙0001] |
| Day_Region 2_4 | -14∙08 [-49∙8 – 21∙65] | -7∙12 [-126∙13 – 111∙89] | 113∙08 [-130∙45 – 356∙61] |
| Day_Region 2_5 | 11∙58 [-7∙57 – 30∙73] | 15∙65 [-48∙86 – 80∙16] | -53∙01 [-184∙93 – 78∙91] |
| Day_Region 2_6 | -3∙38 [-7∙27 – 0∙51] | -5∙57 [-18∙78 – 7∙64] | 9∙13 [-17∙91 – 36∙16] |
| Day_Region 3_1 | -0∙04 [-0∙07 – -0∙02] | -0∙06 [-0∙16 – 0∙05] | -0∙07 [-0∙27 – 0∙13] |
| Day_Region 3_2 | 0∙003 [0∙0015 – 0∙0045] | 0∙0042 [-0∙0016 – 0∙0099] | 0∙0043 [-0∙0072 – 0∙02] |
| Day_Region 3_3 | -0∙000073 [-0∙0001– -0∙000036] | -0∙000091 [-0∙0002 – 0∙000045] | -0∙000095 [-0∙0004 – 0∙0002] |
| Day_Region 3_4 | 67∙06 [24∙92 – 109∙2] | 74∙83 [-78∙39 – 228∙05] | 83∙32 [-225∙63 – 392∙28] |
| Day_Region 3_5 | -23∙87 [-46∙52 – -1∙23] | -22∙85 [-104∙37 – 58∙68] | -29∙36 [-193∙46 – 134∙74] |
| Day_Region 3_6 | 2∙43 [-2∙18 – 7∙04] | 1∙52 [-14∙95 – 17∙99] | 3∙24 [-29∙81 – 36∙28] |
| Day_Region 4_1 | -0∙0018 [-0,02 – 0,02] | -0∙06 [-0∙15 – 0∙04] | -0∙13 [-0∙29 – 0∙03] |
| Day_Region 4_2 | 0,0004 [-0∙0006 – 0∙0014] | 0∙0045 [-0∙0004 – 0∙0093] | 0∙0089 [0∙0005 – 0∙02] |
| Day_Region 4_3 | -0∙000011 [-0∙000035 – 0∙000013] | -0∙0001 [-0∙0002 – 0] | -0∙0002 [-0∙0004 – -0∙000021] |
| Day_Region 4_4 | 4∙43 [-22∙95 – 31∙81] | 114∙51 [-8∙44 – 237∙46] | 231∙88 [16∙2 – 447∙56] |
| Day_Region 4_5 | 3∙31 [-11∙41 – 18∙03] | -51∙96 [-117∙05 – 13∙12] | -115∙08 [-229∙75 – -0∙4] |
| Day_Region 4_6 | -1∙77 [-4∙78 – 1∙25] | 8∙58 [-4∙59 – 21∙76] | 21∙59 [-1∙73 – 44∙9] |
| Day_Region 5_1 | -0∙0006 [-0∙01 – 0∙01] | -0∙0019 [-0∙05 – 0∙04] | -0∙03 [-0∙11 – 0∙05] |
| Day_Region 5_2 | 0∙0004 [-0∙0002 – 0∙001] | -0∙0004 [-0∙0028 – 0∙002] | 0∙0004 [-0∙004 – 0∙0049] |
| Day_Region 5_3 | -0∙000016 [-0∙00003 – -0∙000001] | 0∙000012 [-0∙000045 – 0∙000069] | -0∙000006 [-0∙0001 – 0∙0001] |
| Day_Region 5_4 | 10∙4 [-6∙02 – 26∙82] | -22∙38 [-85∙68 – 40∙93] | -0∙33 [-119∙55 – 118∙89] |
| Day_Region 5_5 | 2∙25 [-6∙38 – 10∙87] | 19∙58 [-13∙57 – 52∙74] | 5∙79 [-57∙25 – 68∙84] |
| Day_Region 5_6 | -2∙19 [-3∙92 – -0∙47] | -5∙61 [-12∙22 – 0∙99] | -2∙35 [-15∙01 – 10∙32] |
| Day_Region 6_1 | 0∙02 [0∙0061 – 0∙03] | 0∙01 [-0∙03 – 0∙06] | 0∙05 [-0∙04 – 0∙13] |
| Day_Region 6_2 | 0∙000003 [-0∙0006 – 0∙0006] | 0∙0004 [-0∙0019 – 0∙0028] | -0∙001 [-0∙0056 – 0∙0036] |
| Day_Region 6_3 | -0∙000016 [-0∙000029 – -0∙000002] | -0∙000028 [-0∙000083 – 0∙000026] | -0∙000006 [-0∙0001 – 0∙0001] |
| Day_Region 6_4 | 19∙64 [4∙88 – 34∙39] | 36∙88 [-23∙4 – 97∙16] | 21∙83 [-96∙3 – 139∙95] |
| Day_Region 6_5 | -6∙89 [-14∙69 – 0∙91] | -17∙55 [-49∙17 – 14∙08] | -13∙61 [-75∙89 – 48∙68] |
| Day_Region 6_6 | 0∙38 [-1∙19 – 1∙95] | 2∙82 [-3∙5 – 9∙15] | 2∙69 [-9∙83 – 15∙21] |
| Day_Region 7_1 | -0∙03 [-0∙05 – -0∙0063] | 0∙06 [-0∙05 – 0∙16] | 0∙23 [0∙00 – 0∙46] |
| Day_Region 7_2 | 0∙0011 [-0∙0003 – 0∙0024] | -0∙002 [-0∙0077 – 0∙0036] | -0∙01 [-0∙03 – -0∙00] |
| Day_Region 7_3 | -0∙000027 [-0∙000058 – 0∙000004] | 0∙000023 [-0∙0001 – 0∙0002] | 0∙0003 [0∙000013 – 0∙0006] |
| Day_Region 7_4 | 28∙38 [-6∙76 – 63∙52] | -13∙74 [-157∙89 – 130∙42] | -332∙58 [-654∙95 – -10∙22] |
| Day_Region 7_5 | -11∙86 [-30∙57 – 6∙86] | 6∙34 [-69∙70 – 82∙38] | 176∙45 [4∙89 – 348∙02] |
| Day_Region 7_6 | 1∙58 [-2∙21 – 5∙37] | -1∙60 [-16∙9 – 13∙71] | -35∙81 [-70∙61 – -1∙01] |
| Age 25-29 | 0∙16 [0∙14 – 0∙19] | 0∙39 [0∙20 – 0∙58] | 0∙16 [-0∙71 – 1∙04] |
| Age 30-34 | 0∙19 [0∙17 – 0∙21] | 0∙75 [0∙57 – 0∙92] | 0∙96 [0∙21 – 1∙71] |
| Age 35-39 | 0∙14 [0∙11 – 0∙16] | 1∙01 [0∙84 – 1∙18] | 1∙38 [0∙66 – 2∙10] |
| Age 40-44 | 0∙15 [0∙13 – 0∙18] | 1∙30 [1∙13 – 1∙46] | 2∙14 [1∙45 – 2∙84] |
| Age 45-49 | 0∙16 [0∙14 – 0∙19] | 1∙64 [1∙48 – 1∙81] | 2∙68 [2∙00 – 3∙36] |
| Age 50-54 | 0∙13 [0∙10 – 0∙15] | 1∙93 [1∙77 – 2∙09] | 2∙99 [2∙31 – 3∙66] |
| Age 55-59 | 0∙06 [0∙03 – 0∙08] | 2∙23 [2∙07 – 2∙39] | 3∙63 [2∙97 – 4∙30] |
| Age 60-64 | -0∙13 [-0∙16 – -0∙10] | 2∙34 [2∙18 – 2∙50] | 4∙01 [3∙34 – 4∙67] |
| Age 65-69 | -0∙24 [-0∙28 – -0∙2] | 2∙53 [2∙37 – 2∙69] | 4∙50 [3∙84 – 5∙16] |
| Age 70-74 | -0∙27 [-0∙32 – -0∙23] | 2∙78 [2∙60 – 2∙95] | 4∙76 [4∙09 – 5∙43] |
| Age 75-79 | -0∙25 [-0∙31 – -0∙2] | 3∙04 [2∙86 – 3∙21] | 5∙26 [4∙59 – 5∙93] |
| Age 80-84 | -0∙19 [-0∙26 – -0∙13] | 3∙32 [3∙14 – 3∙50] | 5∙67 [5∙00 – 6∙34] |
| Age 85+ | -0∙18 [-0∙25 – -0∙11] | 3∙51 [3∙33 – 3∙69] | 6∙05 [5∙39 – 6∙72] |
| Men | -0∙05 [-0∙06 – -0∙04] | 0∙36 [0∙31 – 0∙40] | 0∙57 [0∙48 – 0∙66] |
| Monday | -0∙19 [-0∙22 – -0∙17] | -0∙06 [-0∙15 – 0∙03] | 0∙16 [-0∙01 – 0∙34] |
| Tuesday | 0∙23 [0∙21 – 0∙26] | 0∙19 [0∙11 – 0∙27] | 0∙24 [0∙07 – 0∙41] |
| Wednesday | 0∙48 [0∙46 – 0∙50] | 0∙27 [0∙19 – 0∙35] | 0∙32 [0∙15 – 0∙49] |
| Thursday | 0∙45 [0∙43 – 0∙48] | 0∙26 [0∙18 – 0∙34] | 0∙28 [0∙11 – 0∙45] |
| Friday | 0∙39 [0∙37 – 0∙41] | 0∙19 [0∙10 – 0∙27] | 0∙22 [0∙04 – 0∙39] |
| Saturday | 0∙27 [0∙25 – 0∙29] | 0∙05 [-0∙04 – 0∙14] | 0∙19 [0∙01 – 0∙36] |
|  |  |  |  |
| Intercept | -8∙73 [-8∙81 – -8∙64] | -13∙28 [-13∙63 – -12∙93] | -16∙40 [-17∙32 – -15∙49] |
|  |  |  |  |
| $\rho_{1}$ | 0∙59 [0∙53 – 0∙64] | 0∙76 [0∙68 – 0∙85] | 0∙63 [0∙47 – 0∙80] |
| $\rho_{2}$ | 0∙93 [0∙90 – 0∙96] | 1∙00 [0∙97 – 1∙00] | 1∙00 [0∙97 – 1∙00] |

**Model fit and overdispersion**

We checked the overall model fit and estimated the overdispersion parameter (sections 6.2.3 and 6.2.4 in ^1^) by computing

$T=\sum_{a,s,d,AS} (Y_{a,s,d,AS}-\hat{Y}_{a,s,d,AS})^{2}/\hat{Y}_{a,s,d,AS}$,

where $\hat{Y}_{a,s,d,AS}$is the estimate of $E(Y_{a,s,d,AS})$ based on p=169 parameters, including effectiveness parameters. If the model fits perfectly, *T* is distributed approximately as a chi-squared distribution with *n-p* = 482,160-169=481,991 degrees of freedom, where $n$is the number of ecologic units. We found *T*=461,475. The overdispersion parameter was estimated as *T*/481,993=0.96, giving no indication of overdispersion. Therefore, we did not correct our estimates of standard error for overdispersion.

**Maximum likelihood estimates at a boundary**

In most analyses, parameter estimates were at the interior of the parameter space, and confidence were constructed using asymptotic normality with a robust sandwich estimate of variance. In some analyses $\hat{\rho}_{2}$was at the boundary 1.0. In this case we computed the profile likelihood by maximizing the likelihood over $\beta$and $\rho_{1}$for each of a range of values of $\rho_{2},$including $\rho_{2}=1.$ A lower 95% confidence limit for $\rho_{2}$was found by finding that value of $\rho_{2}$such that twice the log profile likelihood at $\rho_{2}$minus twice the log profile likelihood at $\rho_{2}=1$ equals 2.706 (the 90^th^ percentile of a chi-square variate with one degree of freedom), as discussed on pages 224-227 in reference^2^.

**Estimation of the total number of events prevented by vaccination and its variance**

The estimated total number of events prevented from December 7, 2020 to May 13, 2021 is the sum over ecologic units, including day, of the difference between the numbers of events expected without vaccination minus the numbers expected in the presence of the vaccinations reported, including vaccinations with one or two doses. The estimated value of this difference is

$$=\sum_{a,s,d,AS} \hat{\zeta}_{a,s,d,AS}$$

where the hat symbol indicates a parameter estimate.

Confidence intervals were estimated using a parametric bootstrap with 100 replications. All the parameters, including vaccine efficacies, were assumed to be jointly normal with means given by the original estimates and with covariance obtained by unconstrained maximum likelihood. Then in each bootstrap replication the parameters were resampled and the number of cases/hospitalizations/deaths averted by vaccination was calculated. If $\hat{\rho}_{2}$exceeded 1.0 in a bootstrap replicate, it was set equal to 1.0. Confidence interval limits on events averted were the 2.5 percentile and 97.5 percentile of the bootstrap distribution.

**Sensitivity analyses**

Here we provide data on the sensitivity of our results to several of the analytic choices used in the main analysis. We set the proportion with one dose in an ecologic unit to be equal to the maximum of the observed proportion with one dose and the observed proportion with two doses, because to have two doses one must have had one dose. When we used the observed proportion with one dose instead, estimates of vaccine effectiveness were hardly affected: for incident cases 1^st^ dose 58% (53-64), 2^nd^ dose 92% (89-95); for hospitalizations 1^st^ dose 76% (68-85), 2^nd^ dose 100% (97-100); and for deaths 1^st^ dose 62% (45-79), 2^nd^ dose 100% (97-100). In the main analysis, we set the population size in an age x gender x *AS* group equal to the maximum of the census estimate and the number of vaccinees reported, and we reduced the population size in other such groups proportionally to preserve overall census totals. If we do not reduce size in the other groups, estimates of vaccine effectiveness were hardly affected: for incident cases 1^st^ dose 59% (53-65), 2^nd^ dose 93% (90-96); for hospitalizations 1^st^ dose 78% (69-86), 2^nd^ dose 100% (97-100); and for deaths 1^st^ dose 64% (48-81), 2^nd^ dose 100% (97-100).

We previously analyzed nationwide surveillance data from December 1, 2020 to May 13, 2021. When we changed the starting date to February 1, 2021, shortly after vaccinations were being reported (Figure 1), we obtained very similar estimates of vaccine effectiveness after 2 doses as found in Table 2 of the text: 91% (88-95) for incident cases, 98% (93-100) for hospitalizations, and 99% (93-100) for deaths. Effectiveness estimates after one dose were lower than in Table 2 of the text: 57% (51-63) for incident cases, 66% (55-78) for hospitalizations, and 45% (20-69) for deaths). Because few hospitalizations and deaths occur between the first and second dose, the estimates of efficacy for hospitalizations and deaths for one dose are more uncertain than for two doses, as reflected in the larger confidence intervals for one dose seen above and in Table 2.

**References**

1. McCullagh P, Nelder JA. Generalized Linear Models. Second ed: Taylor & Francis; 1989.

2. Cox DR, Hinkley DV. Theoretical Statistics. London: Chapman and Hall; 1974.
