# Supplementary material for: Estimating vaccine effectiveness against SARS-CoV-2 infection, hospitalization and death from ecologic data in Costa Rica
Source: BMC Infect Dis. 2022 Oct 2;22:767. doi: 10.1186/s12879-022-07740-5 (PMC9526815; doi:10.1186/s12879-022-07740-5)
Supplement: Supplementary file 2 — Additional file 2. Other members of the RESPIRA Study Group [file 12879_2022_7740_MOESM2_ESM.docx]

**Additional File 2**

Members of the RESPIRA Study Group who did not contribute directly to this paper.

Caja Costarricense de Seguro Social: Arturo Abdelnour^7^, Alejandro Calderón^7^, Karla Moreno^7^, Melvin Morera^7^, Roy Wong^7^.
Ministerio de Salud, Costa Rica: Roberto Castro^8^.
Agencia Costarricense de Investigaciones Biomédicas – Fundación INCIENSA, Costa Rica: Bernal Cortés^9^, Viviana Loría^9^, Rebecca Ocampo^9^, Carolina Porras^9^, Michael Zúñiga^9^.
Fogarty International Center, National Institutes of Health: Kaiyuan Sun^10^.

^7^Caja Costarricense de Seguro Social, San José, Costa Rica.  ^8^Ministerio de Salud, San José, Costa Rica. ^9^Agencia Costarricense de Investigaciones Biomédicas – Fundación INCIENSA, San José, Costa Rica. ^10^Fogarty International Center, National Institutes of Health, Bethesda, United States of America.
